# Multilayered salt water with high optical transparency for EMI shielding applications

**DOI:** 10.1038/s41598-020-78717-0

**Published:** 2020-12-09

**Authors:** Duy Tung Phan, Chang Won Jung

**Affiliations:** Graduate School of Nano IT Design Fusion, Seoul National University of Science and Technology, Seoul, 01811 South Korea

**Keywords:** Engineering, Materials science, Optics and photonics

## Abstract

Electromagnetic interference (EMI) shielding for visual observation applications, such as windows utilized in military or aerospace, is important but difficult to realize due to conventional materials having difficulty in achieving sufficient transparency and EMI shielding simultaneously. In this paper, we present multilayered structures based on salt water for simultaneous highly optical transparency (OT) and EM shielding effectiveness (SE) performance. In the proposed structures, planar acrylic and glass were used as two types of clear substrates to hold salt water. The measured OT of both acrylic/salt water/acrylic and glass/salt water/glass structures was higher than 90% with a nearly uniform light transmission, which introduced a negligible impact on optical observation. Furthermore, both simulations and experimental results demonstrated that the SE of the multilayer structure was higher than 20 dB in the X-band from 7.5 to 8.5 GHz. Moreover, the SE was significantly enhanced by increasing the thickness of the salt water layer. Especially, both OT and SE of the multilayered structures were improved simultaneously by increasing the salinity of the salt water. These proposed structures demonstrate great potential in EMI shielding observation applications.

## Introduction

With the successful development of electronics and wireless communication technology, electromagnetic interference (EMI) from microwave radiation is becoming a new and serious global pollutant source that affects health, the military, factories, and commerce^1–4^. Thus, EMI shielding has assumed vital importance^5^. However, the use of EMI shielding is problematic in applications where visual observation is necessary, such as in the optical windows utilized in military and aerospace equipment^5,6^. Therefore, it is highly desirable to develop a transparent and efficient shielding material/structure.

Over the past few decades, transparent conductive thin films (TCF) have been considered as optimum shielding materials given their optical transparency and favorable EMI shielding performance. For example, indium tin oxide (ITO) has dominated the TCF market owing to its high transparency (85%) and low sheet resistance (10 Ω/sq)^7^. However, ITO has various limitations related to the material itself, such as its fragility and high cost, owing to its crystalline structure and its rare-earth indium component.

Recently, transparent electrodes (TEs) such as graphene^8^, graphene-polymer composites^9^, carbon nanotubes (CNTs)^10^, metallic nanowires (MNWs)^11^ and metallic mesh film (MMF)^4,7^ have studied as alternates to ITO. However, graphene and CNTs cannot achieve high transparency and strong EMI shielding simultaneously given that they are carbon-based materials^12,13^. It has also been reported that MNWs have high levels of optical haze, resulting in low transparency of the TE and the considerable contact resistance between the wires, leading to low shielding performance^14^. MMF can be used with printing technology to achieve low contact resistance between the wires, though stray light caused by diffraction superposition degrades the imaging quality^15,16^. Therefore, developing a transparent EMI shielding material or structure that exhibits all of the aforementioned excellent features simultaneously remains a significant technical challenge.

In this paper, we report a strategy by which to design an optically transparent multilayered structure using salt water that achieves excellent transmittance, covering a wide optical region and displaying typical EMI shielding performance. Moreover, the optical and electrical performance capabilities are tunable by controlling the salinity and thickness of the salt-water layer. To the best of our knowledge, no study has used salt water for electromagnetic shielding applications thus far.

## Optical properties

### Theoretical analysis

As in most transparent media, light is attenuated by reflection at the surface and by internal absorption. Therefore, the transmission of the light through media is defined by the following simple relationship^17^:

1 $$T=1-A-R$$

Here, *T, A,* and *R* are the transmission, absorption, and reflection coefficients, respectively. Therefore, the optical transparency (OT) of a medium can be determined from *A* and *R*. In this section, we present a means by which to determine *A* and *R* of a multilayer structure based on salt water.

**Figure 1:**
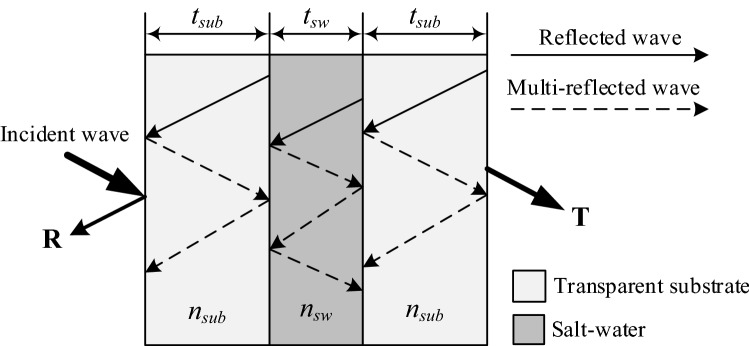
Schematic of the transparent multilayer based on salt water showing the reflections.

Figure 1 shows a schematic (cross-section view) of the optically transparent multilayer structure based on salt water. The multilayer structure consists of a salt-water layer with thickness *t*_*sw*_*,* which is held between two identical transparent substrate layers with thickness *t*_*sub*_. The refractive indexes of the substrate and salt water are ${n}_{sub}$ and ${n}_{sw}$, respectively.

In the multilayer structure, the mechanism of reflection is more complicated than those of single-layer structures owing to the multiple-reflection phenomena. As depicted in Fig. 1, the first reflection is the reflected wave from the transparent material surface, whereas multi-reflection arises in the form of re-reflection of waves reflected earlier. Typically, multiple reflections between the parallel surfaces of a multilayer structure interfere with each other, resulting in the net transparency of the structure being reduced. However, interference only occurs when the spacing between the surfaces is comparable to or narrower than the wavelength of light, which is a few micrometers. In the proposed multilayer structure, the thickness of each layer is several millimeters, which is much wider than the wavelength of light; therefore, the interference due to multiple reflections can be ignored. Accordingly, the reflection at this stage is the only first reflection, and it can be determined using Fresnel equations.

Once the light crosses the interface and enters the bulk, it will be absorbed by the transparent material. The amount of absorption in each layer depends on the thickness and properties of the material itself. The absorption of light in each layer can be determined using Beer-Lambert’s law, i.e., ${A}_{s}=\alpha t$, where $\alpha$ is the absorption coefficient and *t* is the thickness of the layer. The total absorption of the light in the multilayer structure can be determined using Eq. (2).

2 $$A=2{a}_{sub}{t}_{sub}+{\alpha }_{sw}{t}_{sw}$$

In this equation, ${\alpha }_{sub}$ and ${\alpha }_{sw}$ are the absorption coefficients of the transparent substrate and salt water, respectively.

In the multilayer structures here, we used acrylic (poly(methyl methacrylate)) and quartz glass as two types of transparent substrates that correspond to acrylic/salt water/acrylic (ASA) and glass/salt water/glass (GSG) structures, respectively. Quartz glass is a very pure transparent substrate in which the absorption coefficient is very close to zero. The absorption coefficient of the acrylic has also been determined experimentally to be approximately 0.1%/cm^16^. When the total thickness of the two substrate layers is 2 mm (each layer 1 mm), the absorption of light in two transparent substrate layers is approximately 0.02%, which is very small. Moreover, as reported in the literature, the average absorption coefficient of light in seawater is 20%/m^18^; therefore, the absorption of light in a salt-water layer with a thickness of 1 mm is 0.02%. According to Eq. (2), the total absorption of light in multilayer structures would be approximately 0.04%, which is very small to the point that it can be ignored. This was also verified experimentally.

**Figure 2:**
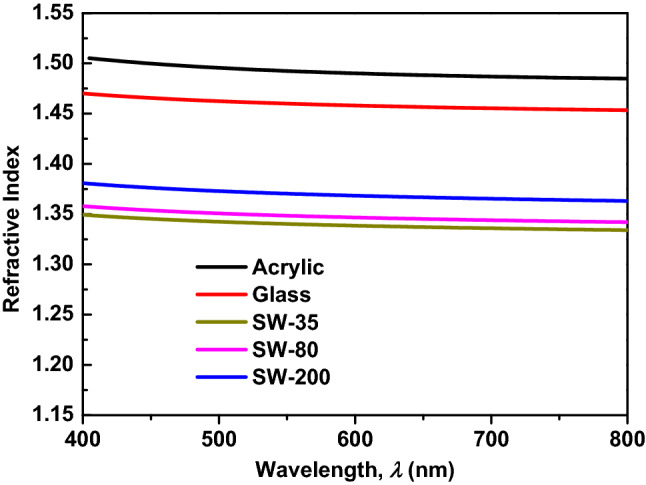
Refractive indexes of transparent substrates versus the wavelength.

Given that the absorption can be ignored, the optical transparency of the multilayer structure depends only on the reflection. Therefore, we analyzed the optical transparency of the multilayer structures based on refractive index matching^19^. Ideal refractive index matching is achieved when two substances with the same refractive index are in contact, causing the light to pass from one substance to the other with neither reflection nor refraction. As shown in Fig. 2, the refractive index of the substrates, in this case the acrylic and glass, is a function of the radiation wavelength^20,21^. It can be observed that both the refractive indexes of acrylic and glass were slightly decreased with an increase in the wavelength. The acrylic showed a significantly higher refractive index compared to that of the glass in the entire visible band.

With regard to salt water, the refractive index depends not only on the light wavelength but also on the salinity, temperature, and salt-water pressure. In relation to this, numerous data sets have been provided through various experiments^1,22–24^. Researchers have reviewed previous studies of the refractive index of salt water and have presented an extensive summary of experimental data as well as interpolations and extrapolations.

In this study, we determined the refractive index of salt water as a function of the salinity and radiation wavelength at room temperature (25 °C) and atmospheric pressure conditions using the empirical equation presented by Quan and Fry^24^

3 $${n}_{sw}={a}_{0}+\left({a}_{1}+{a}_{2}T+{a}_{3}{T}^{2}\right)S+{a}_{4}{T}^{2}+\frac{{a}_{5}+{a}_{6}S+{a}_{7}T}{\lambda }+\frac{{a}_{8}}{{\lambda }^{2}}+\frac{{a}_{9}}{{\lambda }^{3}},$$

where *S* is the salinity in parts per thousand (ppt), *T* is the temperature in degrees Celsius, and $\lambda$ is the radiation wavelength in nanometers. These coefficients are provided by Quan et al.^24^.

Figure 2 shows the refractive index of the salt water as a function of the radiation wavelength ($\lambda$) and salinity at room temperature, i.e., 25 °C. As in the case of acrylic and glass, the index of salt water decreased slightly with an increase in slightly wavelength, especially in the visible band (400–700 nm). Furthermore, the refractive index of salt water increased significantly with an increase in the salinity, and this trend was in good agreement with previous results in the literature^1,22–24^. This analysis of the refractive indexes of the salt water and transparent substrate can be used to explain the measured OT results of the multilayered structures.

## Experimental results

The transparency of the multilayer-based salt water was carefully analyzed in the experiment. OT measurements were conducted using a T60 UV/VIS spectrophotometer. First, the OTs of the transparent substrates (quartz and acrylic) were measured to confirm the measurement accuracy, as their OTs are well known.

**Figure 3:**
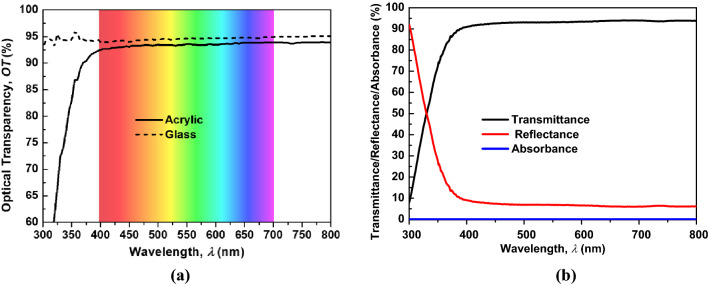
(**a**) Measured OT of transparent substrates and (**b**) measured transmittance, reflectance, and absorbance spectrum of the ASA structure.

Figure 3a shows the measured OTs of acrylic and quartz versus the wavelength, which ranged from 300 to 800 nm. It can be observed that in the visible range (400 to 700 nm), the quartz glass shows a higher OT than acrylic. This result is consistent with the theoretical analysis discussed above, where the refractive index of quartz glass as compared to acrylic is closer to 1 (the refractive index of air); therefore, glass has better refractive index matching with air than with acrylic. The average OTs of the acrylic and quartz glass in the visible range were 93.5% and 94.7%, respectively. Figure 3b shows the measured transmittance, reflectance, and absorbance spectrum of the ASA structure. We found that the absorbance is very small compared to the transmittance and reflectance. This result confirms the theoretical prediction, where the absorbance was ignored.

**Figure 4:**
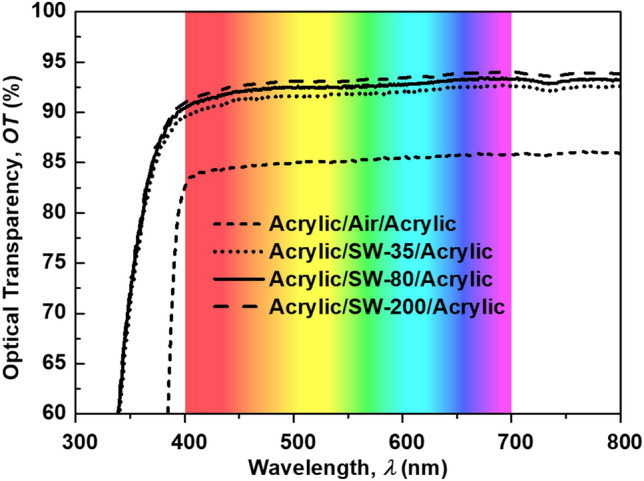
Measured optical transparency of the ASA structure with different salinity levels of salt water in comparison with the OT acrylic/air/acrylic structure.

Figure 4 depicts the OT spectrum of the ASA structure with a different salinity level in comparison with the acrylic/air/acrylic (AAA) structure. It was found that the ASA displayed a higher OT than the AAA structure in the visible band. Moreover, we observed that the OT of the ASA structure increased with an increase in the salinity. This confirmed the above theoretical prediction, which stated that salinity increases lead to an increase in the refractive index and therefore better refractive index matching between the acrylic layers and the salt-water layer.

**Figure 5:**
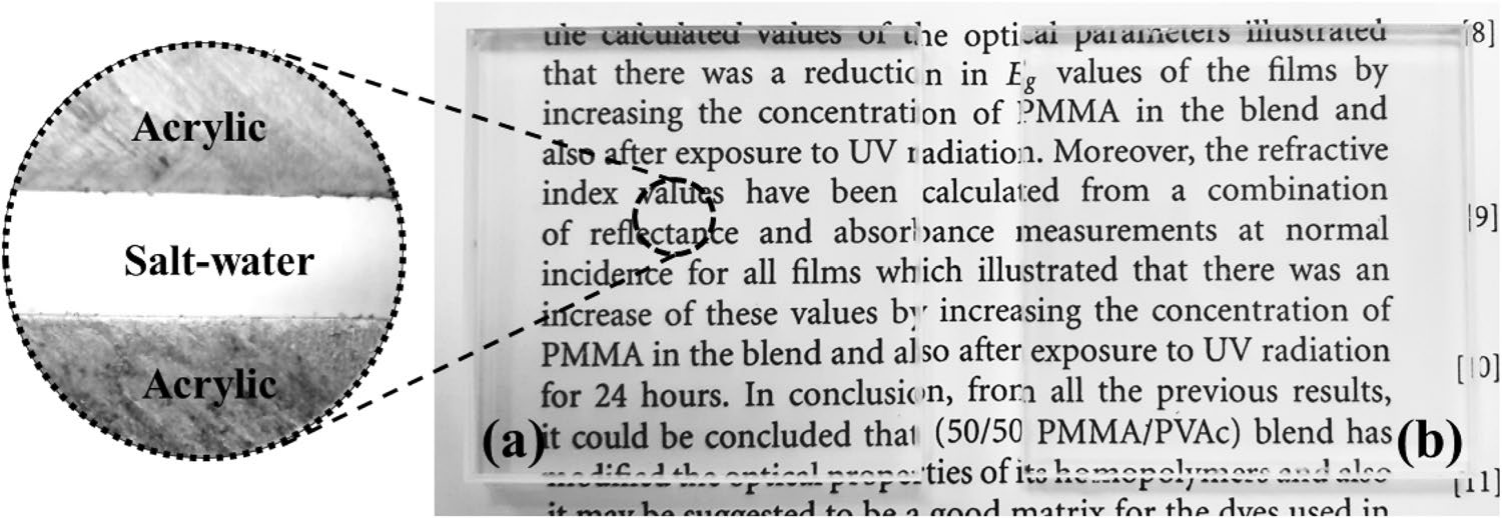
Photograph of the fabricated multilayer-based salt-water samples over text: (**a**) acrylic/salt water/acrylic (front view and cross section view), (**b**) glass/salt water/glass (front view).

**Figure 6:**
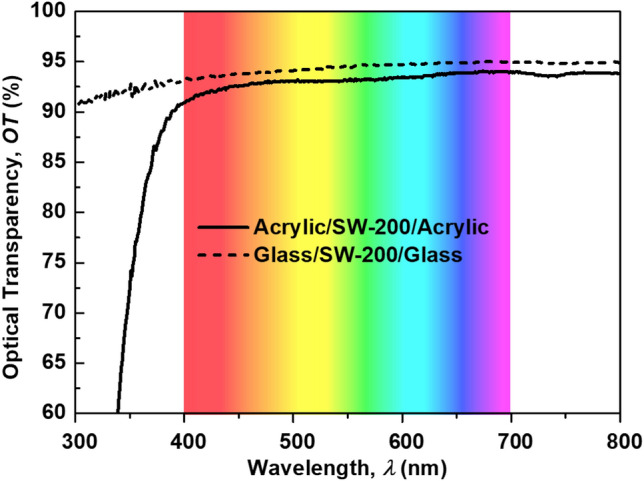
Measured OTs of multilayer structures with different substrates; the salinity of salt water is fixed at 200 ppt.

As shown in Fig. 5a,b, the glass/salt water/glass multilayer structure exhibited high optical transparency, whereas the ASA multilayer structure demonstrated slightly lower transparency. This may be due to the refractive index of the glass being closer to that of salt water than acrylic. Therefore, the GSG structure achieved better refractive index matching than the ASA structure. This can be further proved by the measurement results depicted in Fig. 6. The GSG structure has excellent OT, which can reach 95% with the average value in the visible band being 94.2%. Furthermore, the OT of the ASA structure can reach 94% with the average OT in the visible band being 93.2%.

### EMI shielding of multilayered salt water

#### Electrical characterizations

In this section, we analyze the electrical properties of the multilayered structure in terms of the sheet resistance (*R*_*S*_), which can be determined from the conductivity (σ) and thickness (*t*_*sw*_) of the salt-water layer as ${R}_{s}=1/\left(\sigma {t}_{sw}\right)$. The salt water is an electrolyte solution that produces positive and negative ions that contribute to the conductivity of salt water. Therefore, the conductivity of salt water is a function of the salinity (*S*) and the temperature of the solution. It should be noted that both the ASA and GSG structures have an identical value of *R*_*S*_.

**Figure 7:**
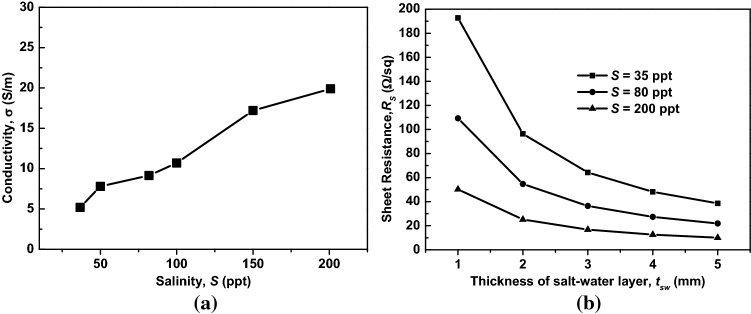
(**a**) Measured conductivity of salt water versus salinity at room temperature and (**b**) sheet resistance of the ASA structure as a function of the salt-water salinity and thickness.

As shown in Fig. 7a, we changed the salinity from 30 to 200 ppt, and it was observed that the conductivity of salt water increased with an increase in the salinity. The measured conductivity levels of the salt water were 5, 10, and 20 S/m, which correspond to salinity levels of 35, 80, and 200 ppt. In actuality, the conductivity of salty water cannot be as high as 100 S/m, as the salt would be saturated in the water. Figure 7b shows the *R*_*S*_ of the multilayered structure as a function of *S* and *t*_*sw*_. It was observed that *R*_*S*_ decreased with an increase in the thickness of the salt-water layer. Moreover, *R*_*S*_ decreased with an increase in the salinity of the salt water due to the increased conductivity, as depicted in Fig. 7a.

In Table 1, we summarize the optical and electrical properties and the figure of merit (FoM) of the proposed structures, i.e., ASA and GSG. Moreover, other TEs from the literature, in this case those of ITO, graphene, carbon nanotubes (CNTs), and silver nanowire, are shown for comparison. In this research, the FoM of each TE is calculated from its sheet resistance and optical transparency using Haacke’s method, as expressed by Eq. (4):

**Table 1:** Summary of OT, Rs and FoM of the proposed structures in comparison with other TEs.

Refs	TE	OT	R_S_	FoM
^7^	ITO	85	10	19.7
^8^	Graphene	97	2100	0.35
^9^	CNTs	70	86	0.32
^13^	Silver nanowire	91	13	30
This work	ASA	94	16.67	32.3
This work	GSG	95	16.67	35.9

4 $$FoM=\frac{O{T}^{10}}{{R}_{S}}$$

The proposed structures together with the graphene and silver nanowire show excellent OTs that exceed 90%, much higher than that of the CNT. Despite the fact that the proposed structures have moderate R_S_, they show the best combination between optical and electrical performance, making their FoM highest among the TEs.

### EMI SE of multilayered structures

The EMI shielding effectiveness (SE) of the transparent multilayered structure was investigated in a simulation and then verified by measurements. First, we simulated the SE of the ASA structure at different salinity levels and thicknesses of the salt-water layer to study the effects of these key parameters on the SE of the multilayered structure. Second, we simulated the SE of the ASA and GSG structures at a salinity level of 200 ppt for a comparison of the two types of substrates. Finally, SE measurements of the ASA and GSG structures at a salinity level of 200 ppt were conducted to verify the simulated results.

**Figure 8:**
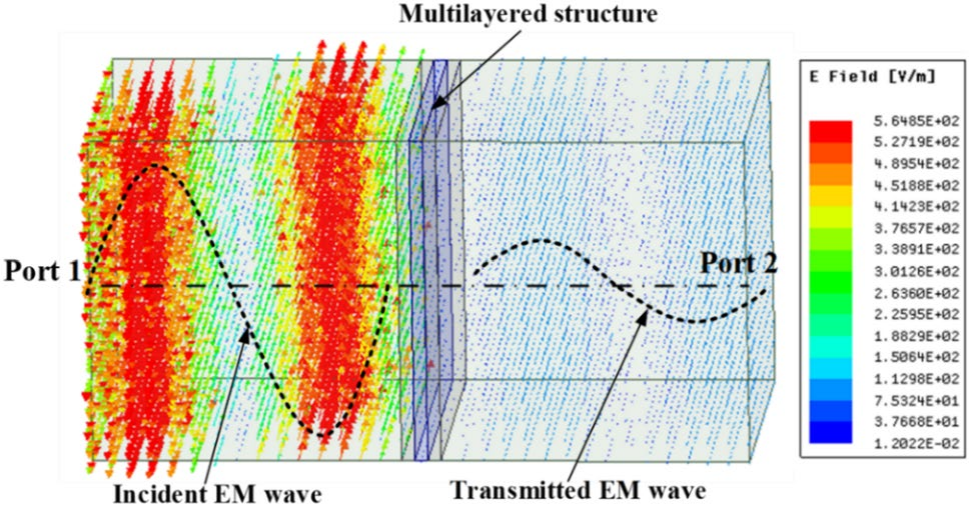
EMI SE simulated schematic of the multilayered structure using Ansys HFSS R 2019.1.

In the simulation, the multilayered structure is placed between two wave ports to determine the power transmission coefficient from port 1 to port 2 (*S*_*21*_), as shown in Fig. 8. We found that the E-field distribution of the incident EM wave was much stronger than the transmitted wave, which confirmed the strong SE of the multilayered structure to block EM waves.

**Figure 9:**
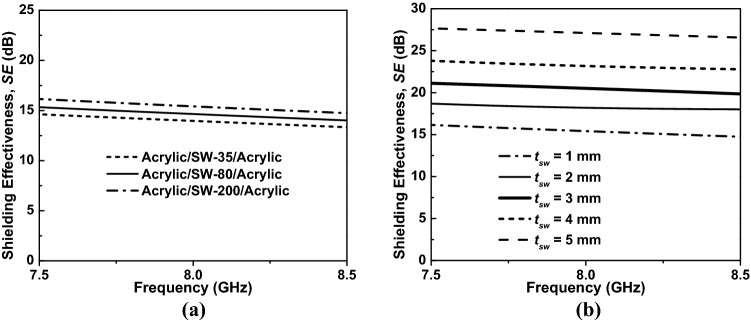
EMI SE of the ASA structure as a function of (**a**) the salinity and frequency and (**b**) the thickness of the salt-water layer and the frequency.

The salinity of the ASA structure changed from 30 to 200 ppt, whereas the thickness of both the acrylic and salt-water layers was fixed at 1 mm. The results revealed that the SE of ASA structure increased as the salinity increased as shown in Fig. 9a. By combining the results in Figs. 4a and 9a, it can be concluded that higher conductivity leads to better shielding performance achieved for the ASA structure.

In Fig. 9b, we investigate the SE of the ASA structure as a function of the salt-water layer thickness in the X-band from 7.5 to 8.5 GHz. In this simulation, we fixed the salinity at 200 ppt and changed the thickness of the salt-water layer to investigate the behavior of the SE. The ASA structure’s EMI SE increased with an increase in the salt-water layer thickness.

**Figure 10:**
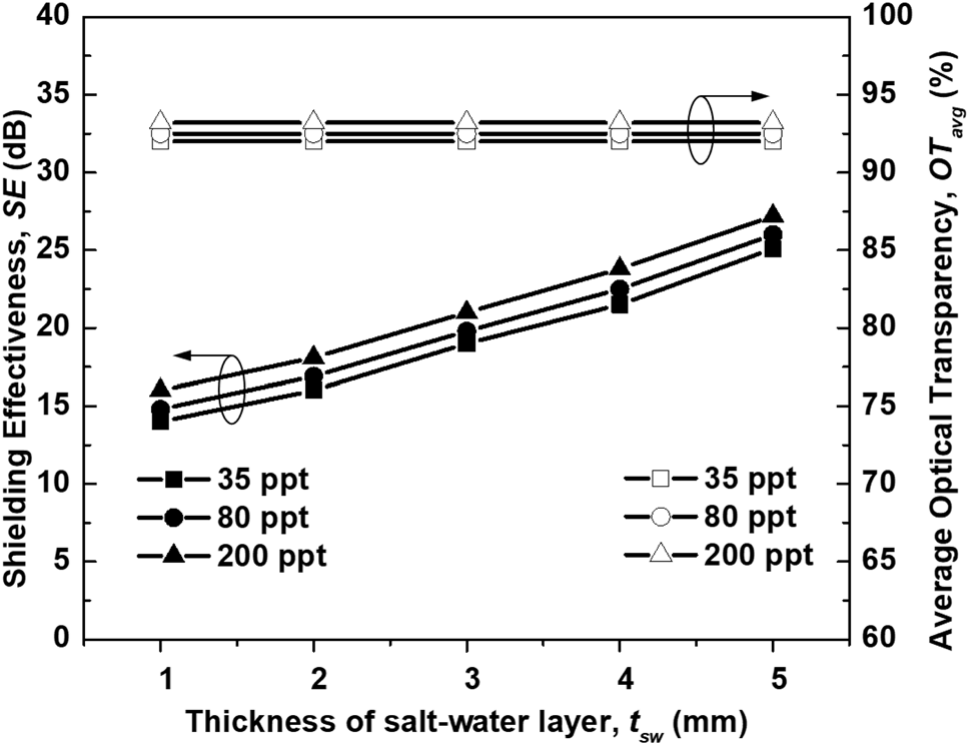
OT and SE of the ASA structure as a function of the salinity and thickness of the salt-water layer.

Finally, the SE and OT of the ASA structure as a function of the salinity and thickness of the salt-water layer are summarized in Fig. 10. The SE was simulated at 8 GHz, whereas the optical performance was the average OT (*OT*_*avg*_) in the visible band with a wavelength ranging from 400 to 700 nm. It was observed that when the salinity increased from 35 to 200 ppt, both the OT_avg_ and SE increased simultaneously. However, the *OT*_*avg*_ and SE of the ASA structure were slightly affected by the variation in the salinity. On the other hand, when the thickness of the salt-water layer varied from 1 to 5 mm, the *OT*_*avg*_ remained nearly unchanged, whereas the SE of the ASA structure significantly improved.

**Figure 11:**
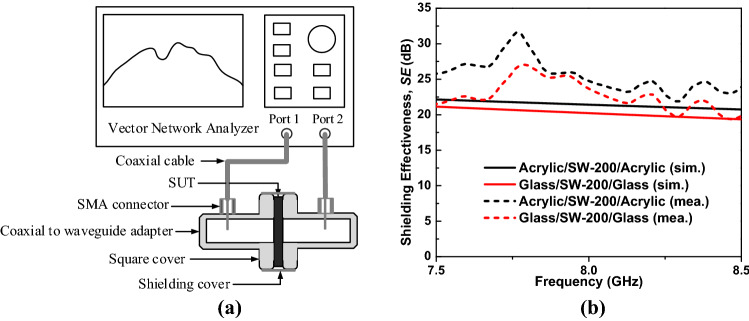
(**a**) SE measurement setup of multilayered structures and (**b**) measured SE of multilayered structures (*S* = 200 ppt, *t*_*sw*_ = 3 mm) in comparison with the simulated results.

To confirm the simulated results, the SE values of the ASA and GSG structures in the X-band frequency range were measured using the setup schematically depicted in Fig. 11a. The sample under test was fabricated using 1-mm-thick acrylic ($\varepsilon =2.7,\mathrm{tan}\delta =0.001$) and 1-mm glass ($\varepsilon =4.3,\mathrm{tan}\delta =0$) as substrates and 200 ppt salt water (@σ = 20 S/m) as the conductive layer (ASA and GSG structures). The total thickness of the multilayered structures was 5 mm; therefore, we used copper tape to block this gap to avoid leakage waves.

The measured and simulated EMI SE outcomes of the transparent multilayered structures are shown in Fig. 11b, showing good agreement between them. The ASA demonstrated a slightly higher SE compared to that of GSG. This can be explained by the loss tangent of the quartz glass, which is close to zero, allowing the EM wave to propagate through without any loss. The ASA with a loss tangent greater than zero also contributes to the total loss of the incident EM wave and therefore introduces a higher SE. The measured SE outcomes of the ASA and GSG structures at 8 GHz were 25.1 dB and 24.1 dB, respectively.

## Conclusion

In conclusion, we demonstrated a high-performance transparent multilayer structure composed of salt water and a clear substrate (acrylic and glass). The experimental results are in good agreement with the theoretical predictions. Specifically, the multilayer structures displayed an average optical transparency (OT) level that exceeded 90% with uniform light transmission and an efficient SE of over 20 dB in the X-band, with salinity of 200 ppt. Moreover, the OT and SE could be tuned by changing the thickness and salinity of the salt-water layer. We found that the SE and the OT increased simultaneously when the salinity of the salt water was increased. Moreover, the SE could be significantly improved by increasing the thickness of the salt-water layer while the OT remained nearly unchanged. With major advantages of a low cost, high transparency, efficient SE, and flexible performance, salt water with the proposed multilayer structure can be considered as a good solution for transparent EMI shielding in visual observation applications.

## Methods

### Materials

We prepare the salt-water solution by dissolving salt in pure water at room temperature (25 °C) in the following two steps: (1) pouring 100 ml of water (@100 g) into a glass beaker, and (2) adding salt to the beaker and stirring it. The amount of salt is calculated to achieve the desired salinity via the relationship of *m*_*s*_ = *100S/(1000—S)*, where *m*_*s*_ is the amount of salt in grams and *S* is the desired salinity of the salt water solution in parts per thousand (ppt). The OT outcomes of the proposed structures were measured using a UV/VIS spectrophotometer (T60 model, PG Instruments Limited Co., UK) connected to a computer.

### Simulation

The EMI SE simulation of the multilayered structure was conducted using the software package Ansys HFSS R 2019.1. As shown in Fig. 8, the multilayered structure is placed between two wave ports to determine the power transmission (*S*_*21*_) from port 1 to port 2. The simulated *SE* is determined from *S*_*21*_ (in dB) as $SE=\left|{S}_{21}\right|$.

### Measurement

The measured σ of the salt water was carried out using a portable electrical conductivity meter (model HI8633, Hanna Instruments Co., US), whereas the *R*_*S*_ values of the proposed structures were determined from the measured $\sigma$ and *t*_*sw*_ as ${R}_{s}=1/\left(\sigma {t}_{sw}\right)$. The experimental EMI SE of the multilayered structures in the X-band was determined using two waveguide-to-coaxial adapters connected to ports 1 and 2 of a vector network analyzer (model E5071B, Keysight Technologies Co., US), as shown in Fig. 11a. The opening cross-section of the adapters was 22.5 mm × 10 mm, corresponding to a bandwidth ranging from 6.5 to 10 GHz. The sample under test (SUT) was identically in size to the outer part (cover flange) of the adapters (45 mm × 45 mm). The SUT was fabricated using 1-mm-thick acrylic ($\varepsilon =2.7,\mathrm{tan}\delta =0.001$) and quartz glass ($\varepsilon =4.3,\mathrm{tan}\delta =0$) as substrates and 3-mm-thick salt water ($S=200 ppt @\sigma =20 S/m$) as the conductive layer (corresponding to the ASA and GSG structures). The total thickness of the SUT was 5 mm; therefore, we used copper tape to block the gap to avoid leakage waves. The measurement procedure of the SUT had two steps: (1) connecting the two adapters directly and measuring the transmission coefficient *S*_*210*_ and (2) separating the two adapters by the SUT and measuring the transmission coefficient *S*_*21S*_. The measured SE of the SUT was determined from the measured transmission coefficients as $SE=\left|{S}_{21S}\right|-\left|{S}_{210}\right|$.
