# Author Correction: Multilayered salt water with high optical transparency for EMI shielding applications

**DOI:** 10.1038/s41598-021-87512-4

**Published:** 2021-04-06

**Authors:** Duy Tung Phan, Chang Won Jung

**Affiliations:** Graduate School of Nano IT Design Fusion, Seoul National University of Science and Technology, Seoul, 01811 South Korea

Correction to: *Scientific Reports* 10.1038/s41598-020-78717-0, published online 09 December 2020

This Article contains errors in Figure 3a, Figure 4, and Figure 6 where the visible light spectrum chart in the background is incorrect (the colours of the light are inversely compared to the corresponded wavelengths).

**Figure 1:**
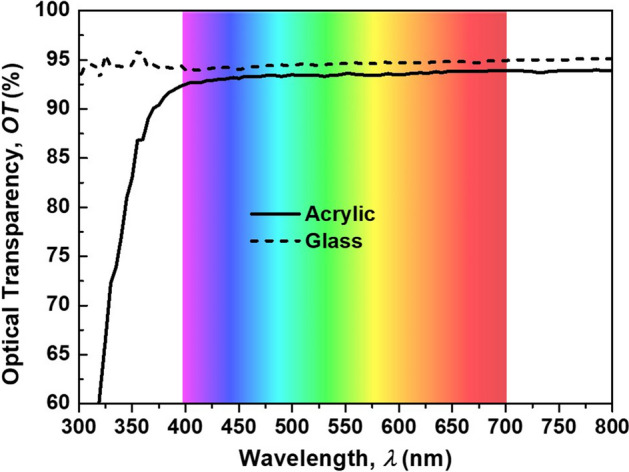
A correct version of the original Figure 3a.

**Figure 2:**
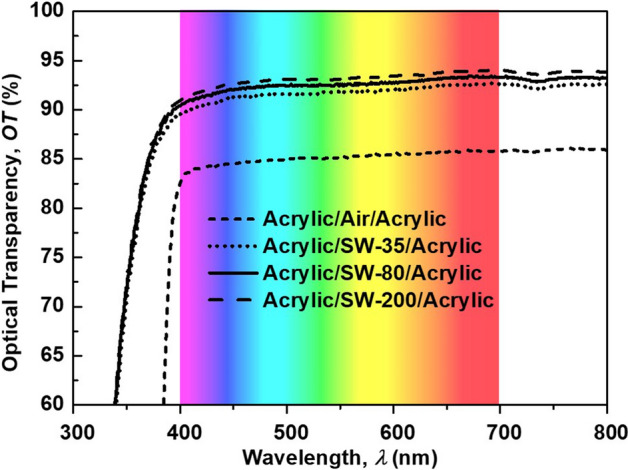
A correct version of the original Figure 4

**Figure 3:**
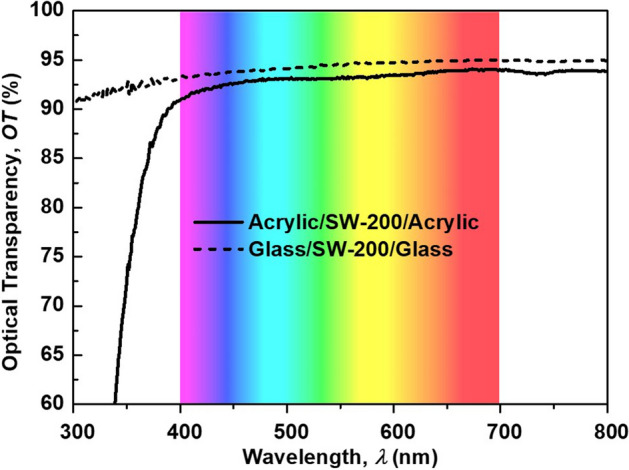
A correct version of the original Figure 6

The correct Figure 3a, Figure 4, and Figure 6 appear below.

